# Haemosporidioses in wild Eurasian blackbirds (*Turdus merula*) and song thrushes (*T. philomelos*): an in situ hybridization study with emphasis on exo-erythrocytic parasite burden

**DOI:** 10.1186/s12936-020-3147-6

**Published:** 2020-02-12

**Authors:** Tanja Himmel, Josef Harl, Simone Pfanner, Nora Nedorost, Norbert Nowotny, Herbert Weissenböck

**Affiliations:** 1 Department for Pathobiology, Institute of Pathology, University of Veterinary Medicine Vienna, Veterinärplatz 1, 1210 Vienna, Austria; 2 Department for Pathobiology, Institute of Virology, University of Veterinary Medicine Vienna, Vienna, Austria; 3 Department of Basic Medical Sciences, College of Medicine, Mohammed Bin Rashid University of Medicine and Health Sciences, Dubai, United Arab Emirates

**Keywords:** *Plasmodium*, *Haemoproteus*, *Leucocytozoon*, Exo-erythrocytic stages, Megalomeront, Chromogenic in situ hybridization, Virulence, *Plasmodium matutinum*, *Plasmodium vaughani*

## Abstract

**Background:**

Passerine birds are frequently infected with diverse haemosporidian parasites. While infections are traditionally considered benign in wild birds, recent studies demonstrated mortalities of passerine species due to exo-erythrocytic development of the parasites, which can damage organs in affected hosts. However, exo-erythrocytic development remains insufficiently investigated for most haemosporidian species and thus little is known about the virulence of tissue stages in wild passerine birds. The aim of the present study was to investigate natural haemosporidian infections in deceased Eurasian blackbirds (*Turdus merula*) and song thrushes (*Turdus philomelos*) and to determine parasite burden and associated histological effects.

**Methods:**

For molecular analysis, blood and tissue samples from 306 thrushes were screened for *Plasmodium*, *Haemoproteus* and *Leucocytozoon* parasites by nested PCR. For the detection of parasite stages in organ samples, tissue sections were subjected to chromogenic in situ hybridization (CISH) using genus- and species-specific probes targeting the rRNAs of parasites. Exo-erythrocytic parasite burden was semi-quantitatively assessed and histological lesions were evaluated in haematoxylin–eosin-stained sections.

**Results:**

By PCR, 179 of 277 Eurasian blackbirds and 15 of 29 song thrushes were positive for haemosporidians. Parasites of all three genera were detected, with *Plasmodium matutinum* LINN1 and *Plasmodium vaughani* SYAT05 showing the highest prevalence. CISH revealed significant differences in exo-erythrocytic parasite burden between lineages in Eurasian blackbirds, with *P. matutinum* LINN1 frequently causing high exo-erythrocytic parasite burdens in various organs that were associated with histological alterations. Song thrushes infected with *P. matutinum* LINN1 and birds infected with other haemosporidian lineages showed mostly low exo-erythrocytic parasite burdens. Two Eurasian blackbirds infected with *Leucocytozoon* sp. TUMER01 showed megalomeronts in various organs that were associated with inflammatory reactions and necroses.

**Conclusion:**

This study suggests that *P. matutinum* LINN1, a common lineage among native thrushes, regularly causes high exo-erythrocytic parasite burdens in Eurasian blackbirds, which may result in disease and mortalities, indicating its high pathogenic potential. The findings further illustrate that the same parasite lineage may show different levels of virulence in related bird species which should be considered when assessing the pathogenicity of haemosporidian parasite species. Finally, the study provides evidence of virulent *Leucocytozoon* sp. TUMER01 infections in two Eurasian blackbirds caused by megalomeront formation.

## Background

Avian haemosporidians compose a highly diverse group of apicomplexan parasites that occur almost globally and infect a wide range of different bird species from most families [1]. The three major genera *Plasmodium*, *Haemoproteus,* and *Leucocytozoon* comprise more than 200 morphologically described species [1] and almost 4000 unique mitochondrial cytochrome b (*cytb*) lineages have been recorded to date [2].

Wild passerine birds (Passeriformes) are commonly infected with haemosporidian parasites belonging to all three genera [3] and more than one-third of all described haemosporidian species have been found in this bird order [1, 4]. The parasites harboured by passerine birds vary greatly in prevalence and their level of host-specificity, ranging from generalists, which infect a wide range of passerine species, to extreme host-specialists which confine to single species [5–8]. Haemosporidian infections in passerine birds are usually considered benign, as individuals are often chronically infected exhibiting low levels of parasitaemia and rarely showing signs of illness [1, 9]. The observed low virulence is explained by evolutionary reciprocal adaption of birds and parasites over long time. In contrast, infection of immunologically naïve host species regularly results in severe morbidity and has caused mortalities in several non-passerine bird species [10–18]. However, several experimental infection studies demonstrated also high susceptibility of passerine bird species to certain haemosporidian parasites and markedly different pathogenicity depending both on infected host species and parasite strain [19–23]. Moreover, few reports showed lethal infections with *Plasmodium* spp. in wild passerine birds which commonly harbour haemosporidian parasites [24–26].

During infection, the parasites undergo complex development in the bird host, which includes several generations of asexual replication in tissue cells (exo-erythrocytic merogony) and invasion of blood cells with gametocyte formation resulting in parasitaemia, which causes haemolysis and manifests in anaemia. Notably, in the above-mentioned cases, pathology was largely induced by severe burdens of exo-erythrocytic stages (meronts) in endothelial cells and other tissue cells in various organs. The detailed pattern of exo-erythrocytic merogony may vary substantially across both parasite and infected host species and remains unknown for the majority of haemosporidian species, particularly from the genera *Haemoproteus* and *Leucocytozoon* [27], because these life stages are difficult to access, especially in wild birds. Together with host-related factors, which presumably influence parasite development, this renders general predictions about their pathogenic impact and virulence in diverse bird hosts difficult.

Traditionally, exo-erythrocytic parasite stages are examined by histological techniques, but their detection is cumbersome because the stages are often small and easily confounded with cellular structures or debris. In order to facilitate the detection in tissue sections and identification of parasites belonging to the three major genera, molecular probes have been developed [14, 28].

The present study intended to investigate avian haemosporidian infections in a large collection of Eurasian blackbirds (*Turdus merula*) and song thrushes (*Turdus philomelos*) which were found dead in Vienna and suburban regions. This investigation extends earlier research, which showed an association of *Plasmodium* spp. infections with mortalities of several passerine species native to Austria [25] and aims to determine the burden of *Plasmodium*, *Haemoproteus* and *Leucocytozoon* parasites in tissue samples of deceased birds and to evaluate their histological impact by chromogenic in situ hybridization, a combined molecular-histological approach.

## Methods

### Samples

Blood and tissue samples of 306 thrushes (Turdidae, Passeriformes) including 277 blackbirds (*Turdus merula*) and 29 song thrushes (*T. philomelos*) were obtained from the Institute of Pathology at the University of Veterinary Medicine Vienna (Austria). The samples were collected during post-mortem examinations of dead birds submitted between 2002 and 2018. Most birds were found between July and October with the majority being collected in August. Fifteen birds were collected between November and March. The birds were found in different parts of Vienna and surrounding districts of the Federal State of Lower Austria, few birds originated from the neighbouring countries Switzerland and Hungary. A small number of the samples was already analysed earlier by Dinhopl et al. [25], however, they were screened using the primers Palu-F and Palu-R by Martínez et al. [29], which do not allow detection of *Leucocytozoon* parasites. In order to re-evaluate these samples using a current standard PCR assay [30] and recently developed molecular probes [28], they were included in this study as well. Tissue samples were taken from several organs, including heart, lung, liver, spleen, kidney, brain, gizzard and intestine in most cases and in some also skeletal muscle and bursa of Fabricius. For histology and chromogenic in situ hybridization (CISH) of tissue sections, samples were fixed in formalin and embedded in paraffin wax (FFPE). For molecular analysis, tissue samples were frozen and stored at − 80 °C until further use.

### Molecular analysis

DNA was extracted from organ homogenates of 298 birds [31] and from dried blood spots of eight birds using the DNeasy Blood & Tissue Kit (Qiagen, Venlo, Netherlands) following the manufacturer’s recommendations with the exception that two elution steps with each 100 µl AE buffer were performed, the second of which was used as template for PCR. For detection of haemosporidians, a nested PCR protocol [30] targeting a section of the mitochondrial cytochrome b gene (*cytb*) of parasites was used. First, the primers HaemNFI and HaemNR3 were used to amplify DNA of *Plasmodium*, *Haemoproteus* and *Leucocytozoon*. For the second PCR, the primer pairs HaemF/HaemR2 and HaemFL/HaemR2L were used to amplify DNA of *Plasmodium*/*Haemoproteus,* and *Leucocytozoon*, respectively.

PCR was performed in 25 µl reaction volumes containing 14.375 µl nuclease-free water, 5 µl 5× Green GoTaq Flexi Buffer (Promega, Madison, Wisconsin, USA), 2 µl MgCl_2_ solution (25 mM), 0.5 µl PCR nucleotide mix (10 mM, Promega), 0.125 µl GoTaq G2 Flexi DNA Polymerase (5 u/µl, Promega), each 1 µl forward and reverse primer (10 pmol/µl), and 1 µl of DNA template. For the second PCRs, instead of a DNA template, 1 µl amplicon from the first PCR was used. Thermocycler conditions included initial denaturation at 94 °C for 2 min, 35 cycles of denaturation at 94 °C for 30 s, primer annealing at 50 °C for 30 s and extension at 72 °C for 1 min, followed by a final extension at 72 °C for 10 min. PCR products were visualized on 1% agarose gels with Midori Green Advance (Nippon Genetics Europe, Dueren, Germany) using a BioSens SC-Series 710 gel documentation system (GenXpress, Wiener Neudorf, Austria). In every PCR run, positive controls (tissue samples confirmed positive in earlier screenings) and negative controls (nuclease free water instead of DNA templates) were included. PCR amplicons from the second PCRs were sent to Microsynth Austria for bi-directional sequencing. Obtained sequences were edited and aligned in Bioedit [32] and electropherograms were carefully checked for ambiguities. In case sequences contained ambiguous characters indicating double infections, electropherograms were carefully re-checked and sequences were un-phased using DnaSP v.6.12.3 [33]. Sequences were subjected to BLAST search using the avian malaria database MalAvi [2] and NCBI GenBank. Haplotypes not showing 100% matches with published *cytb* lineages were given new lineage names according to MalAvi rules.

### Chromogenic in situ hybridization (CISH)

For the detection of parasites in tissue sections, FFPE tissue blocks of each individual bird were cut into 1–2 µm thick sections, one of which was stained with haematoxylin and eosin (HE) and the remaining ones were subjected to CISH. Chromogenic in situ hybridization was performed following previously established protocols using genus-specific oligonucleotide probes for the detection of *Plasmodium*, *Haemoproteus*, and *Leucocytozoon* [14, 28]. In cases of mixed infections, as determined by PCR and sequencing, several sections were separately incubated with all relevant probes. For a detailed analysis of double infections with *Plasmodium vaughani* and *Plasmodium matutinum*, species-specific oligonucleotide probes were designed based on *18S* ribosomal RNA gene sequences published by Harl et al. [34]. These probes were tested on tissue sections from birds infected with the respective parasites (single infections) and cross-reactions were ruled out by negative results. Tissue sections from birds harbouring those two lineages were then separately incubated with the species-specific probes. Sequences of all probes used in this study are listed in Table 1.

**Table 1:** Oligonucleotide probes used for the detection of haemosporidian parasites by chromogenic in situ hybridization

Probe	Sequence	Target	References
Plas18S	5′-TTTAATAACTCGTTATATATATCAGTGTAGCAC-3′	*Plasmodium* spp.	Dinhopl et al. [25]
Haemo18S_1	5′-GCTAACCGTAGTTATAGTCGCCATCTC-3′	*Haemoproteus* (*Parahaemoproteus*) spp.	Himmel et al. [28]
Leuco18S_1	5′-TAGGACTCCCCACTTGTCTTTTTCTTGA-3′	*Leucocytozoon* (*Leucocytozoon*) spp.	Himmel et al. [28]
Pmat18S_1b	5′-CTAAAAGCATCTTGACTGTTACATCAAGACG-3′	*Plasmodium matutinum* (LINN1, AFTRU5)	Present study
Pvau18S_1b	5′-CAAGCCGAAACTAATTACATTTAAACATAAGATCC-3′	*Plasmodium vaughani* (SYAT05)	Present study

For CISH, tissue sections were deparaffinized in Neo-Clear solution (Merck Millipore, Burlington, Massachusetts, USA), rehydrated in a series of graded ethanol (100%, 96%, 70%) and distilled water and pre-treated with proteinase K (Roche, Basel, Switzerland) 3 µg/ml in 0.5 M Tris–HCl buffered saline at 37 °C for 40 min. After proteolysis, sections were rinsed in distilled water, rehydrated in 96% and 100% ethanol and air-dried. For hybridization, tissue sections were incubated with hybridization solution containing 11 µl distilled water, 20 µl 20× saline-sodium citrate (SSC) buffer, 50 µl formamide, 5 µl herring sperm, 2 µl 50× Denhardt’s solution, 10 µl dextran sulphate (50%, w/v) and 1 ng probe per 100 µl overnight in a humid chamber at 40 °C. On the next day, sections were subjected to stringency washes in 2×, 1× and 0.1× SSC buffer 10 min each and pre-incubated with Tris–HCl buffered saline containing normal goat serum and 10% Triton X-100 for 30 min before application of anti-digoxigenin-AP Fab-fragments (Roche) at a concentration of 1:200 for 1 h at room temperature. After washing in distilled water 2 × 15 min, chromogenic detection was accomplished by application of NBT/BCIP (nitro-blue tetrazolium chloride/5-bromo-4-chloro-3′-indolyphosphate *p*-toluidine salt, Roche) mixed with levamisole in 0.1 M Tris-buffered saline (pH 9.5) and incubation for a minimum of 40 min in a dark, humid chamber. Enzymatic reaction was stopped by treating slides with Tris–EDTA buffer (pH 8.0) for 10 min. Finally, sections were counterstained with haematoxylin and mounted using Aquatex (Merck Millipore) and coverslips.

### Histology and evaluation of exo-erythrocytic parasite burden

CISH-treated tissue sections were analysed by bright field microscopy using 100×, 200×, 400× and 1000× magnifications. All organ samples from each bird were examined and the degree of exo-erythrocytic parasite burden was assessed using a semi-quantitative scoring system. Hereby, a clear differentiation was made between CISH signals deriving from infected blood cells, corresponding to erythrocytic stages of parasites, and signals matching exo-erythrocytic stages (tissue meronts). The extent of exo-erythrocytic burden was assessed for each organ sample by a 0 to 3 score system (0 = negative, no meronts detected; 1 = low number of meronts—not in every 200× field; 2 = moderate number of meronts—one or more meronts in several high-power fields (HPF); 3 = high number of meronts—one or more meronts in every HPF). For histological analysis, HE-stained sections were examined.

Microphotographs were acquired using an Olympus BX51 microscope (Olympus Europa, Hamburg, Germany) equipped with an Olympus DP71 camera. Images were adjusted for brightness and contrast and assembled in Adobe Photoshop CC 2019 (Adobe, San José, California, USA).

### Statistics

Analyses were performed using the statistical software package IBM SPSS Statistics Version 24.0 (IBM Corp., Armonk, N.Y., USA). To evaluate for an association between extent of exo-erythrocytic parasite burden and parasite lineage, a Fisher’s exact test was used and calculated for samples sizes > 20. Level of significance was set at 0.05 with *p < 0.05, **p < 0.01 and ***p < 0.001.

## Results

### Molecular analysis

By PCR, 194 (63.4%) of 306 birds were positive for *Plasmodium*, *Haemoproteus*, *Leucocytozoon* or a combination of these genera (Table 2). *Plasmodium* showed the highest prevalence with 177 infected birds, *Haemoproteus* and *Leucocytozoon* were less prevalent with only 15 and 17 positive birds, respectively.

**Table 2:** Prevalence and diversity of haemosporidian parasites recorded in Eurasian blackbirds (*Turdus merula*) and song thrushes (*Turdus philomelos*) by PCR screening

Host species	*n* Examined	PCR positive^a^				Parasite species & *cytb* lineages^b^
		*Plas*	*Haem*	*Leuc*	Total	
*Turdus merula*	277	166/32	12/4	13/11	179/35	*P. matutinum* LINN1 (91/26), AFTRU5 (4/1); *P. vaughani* SYAT05 (88/26); *P. elongatum* GRW06 (1); *Plasmodium* sp. ***TUMER12*** (2), ***TUMER13*** (1), ***TUMER14*** (1/1), ***TUMER15*** (1/1), ***TUMER16*** (1); *H. minutus* TURDUS2 (11/3), TUCHR01 (1/1), ***TUMER17*** (1/1); *Leucocytozoon* sp. TUMER01 (7/7), TUMER10 (1), ASOT06 (3/2), ***AEFUN03*** (1/1), ***TUMER18*** (1/1)
*Turdus philomelos*	29	11/2	3/2	4/4	15/5	*P. matutinum* LINN1 (8/2); *P. vaughani* SYAT05 (1); *P. circumflexum* TURDUS1 (1); *Plasmodium* sp. ***TUPHI08*** (1/1), ***TUPHI09*** (1); *H. minutus* TUPHI01 (3/2); *Leucocytozoon* sp. EUSE2 (2/2), TUPHI06 (1/1), ***TUPHI10*** (2/2), ***TUPHI11*** (1/1), ***TUPHI12*** (1/1)
Total	306	177/34	15/6	17/15	194/40	12 *Plasmodium*, 4 *Haemoproteus*, 10 *Leucocytozoon* lineages

^a^Numbers indicate total infected birds, followed by number of mixed infections

^b^Numbers in parentheses indicate total infected birds, followed by number of mixed infections. Lineages in bolditalic are new *cytB* haplotypes

Of 194 positive birds, 40 (20.6%) showed mixed infections, including 35 double and 5 triple infections. The mixed infection rate was highest among *Leucocytozoon*-positive birds, with 15 (88.2%) of 17 showing a co-infection with either *Plasmodium* (9 cases), *Haemoproteus* (5 cases) or multiple *Leucocytozoon* lineages (1 case). Thirty-four (19.2%) of 177 *Plasmodium*-infected birds harboured multiple *Plasmodium* lineages (24 cases) or were co-infected with *Leucocytozoon* (9 cases) or *Haemoproteus* (1 case). Six (40.0%) of 15 *Haemoproteus*-infected birds showed mixed infections with *Leucocytozoon* (5 cases) or *Plasmodium* (1 case).

By sequencing, 26 unique *cytb* lineages (*Plasmodium*: 12, *Leucocytozoon*: 10, and *Haemoproteus*: 4) were identified, 13 of which were 100% identical to previously reported lineages in MalAvi or Genbank (Table 2).

In blackbirds, the by far most prevalent lineages were LINN1, a lineage of *P. matutinum,* recorded in 91 (50.8%) of 179 infected birds, and SYAT05, a lineage of *P. vaughani,* detected in 88 birds (49.2%). In song thrushes, the prevalence of LINN1 was similar, recorded in eight (53.3%) of 15 infected birds. In contrast, SYAT05 was identified in only one (6.7%) of 15 infected song thrushes. Among blackbirds, four birds featured lineages showing 99% matches with SYAT05 (TUMER13, TUMER14, TUMER15, TUMER16) and one bird featured a 99% match with LINN1 (TUMER12). Two song thrushes featured lineages showing 99% matches with SYAT05 (TUPHI10, TUPHI11). Among *Leucocytozoon*, TUMER01 was most prevalent, detected in seven Eurasian blackbirds. From the genus *Haemoproteus*, the three lineages TUPHI01, TURDUS2 and TUCHR01, all linked to the morphospecies *Haemoproteus minutus*, were identified. One blackbird harboured a sequence (TUMER17) matching 99% with TURDUS2.

### Chromogenic in situ hybridization

All 194 birds (179 Eurasian blackbirds and 15 song thrushes) positive for haemosporidian parasites by PCR and sequencing were analysed by CISH, including 154 single and 40 mixed infections. CISH revealed parasites in tissue sections of 178 cases (91.8%) whereas 16 (8.2%) remained negative. The detected parasite stages were classified as blood or tissue stages based on the shape, size and location of signals. Blood stages typically showed oval to roundish signals not larger than blood cells and were located in capillaries and larger vessels. Signals exceeding the size of blood cells and showing variable shapes were considered exo-erythrocytic meronts (Additional file 1). In unclear cases, e.g., when the signals were all similar and in the size of blood cells, the corresponding HE-stained sections were checked for exo-erythrocytic meronts.

### Detection of *Plasmodium* parasites

CISH detected *Plasmodium* parasites in 165 (93.2%) of 177 PCR-positive birds, but not all birds exhibited exo-erythrocytic stages of the parasites in the tissues. 113 (68.5%) cases showed both erythrocytic and exo-erythrocytic stages of parasites, whereas 46 (27.9%) cases only showed exo-erythrocytic but no blood stages. Conversely, six cases (3.6%) presented blood stages but no exo-erythrocytic meronts.

Exo-erythrocytic parasite burden, i.e. the abundance of tissue stages (hereinafter also referred to as “parasite burden”), varied extensively among individuals, ranging from zero to high numbers of detectable meronts. Representative photographs of low-, moderate- and high-grade parasite burdens are shown for the brain and the spleen (Additional file 2). Of 159 cases that presented exo-erythrocytic stages, 74 (46.6%) exhibited low numbers of meronts in at least one organ, 34 (21.4%) had moderate numbers of meronts in the tissues and 51 (32.1%) showed high burdens of exo-erythrocytic meronts (Table 3). A comparison of exo-erythrocytic parasite burden between infected birds collected in different months shows that birds collected in August were most often affected by high-grade exo-erythrocytic merogony (Additional file 3). Comparing Eurasian blackbirds and song thrushes, the overall percentage of exo-erythrocytic-positive cases was similar with 89.8% (149/166) and 90.9% (10/11), respectively. However, in contrast to blackbirds, which showed various degrees of parasite merogony in their tissues, most song thrushes only exhibited low numbers of exo-erythrocytic meronts.

**Table 3:** *Plasmodium* exo-erythrocytic parasite burden in Eurasian blackbirds (*Turdus merula*) and song thrushes (*Turdus philomelos*) determined by chromogenic in situ hybridization (CISH)

Host species	*n* Examined	Exo-erythrocytic stages detected by CISH				
		Negative	Low^a^	Moderate^a^	High^a^	Total positive
*Turdus merula*	166 (32)	17 (3)	66 (9)	33 (4)	50 (16)	149 (29)
*Turdus philomelos*	11 (2)	1 (0)	8 (1)	1 (0)	1 (1)	10 (2)
Total	177 (34)	18 (3)	74 (10)	34 (4)	51 (17)	159 (31)

Numbers in parentheses indicate mixed infections with either multiple *Plasmodium* lineages or *Haemoproteus* and *Leucocytozoon* lineages

^a^Numbers of cases exhibiting low, moderate, and high exo-erythrocytic parasite burdens in at least one of the examined organs

**Fig. 1:**
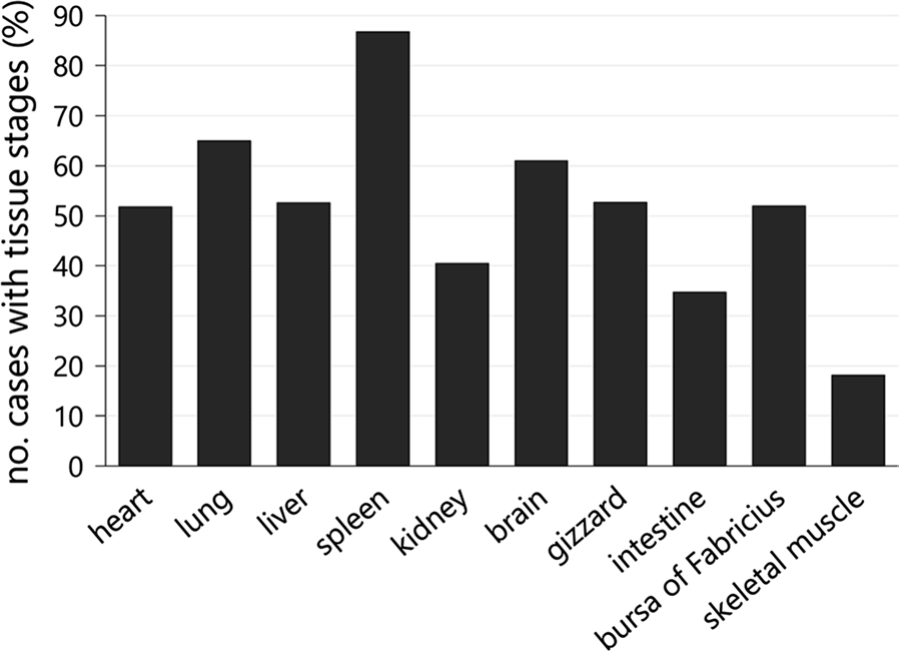
Location of exo-erythrocytic stages of *Plasmodium* spp. in infected Eurasian blackbirds and song thrushes (*n* = 177). The spleen was most commonly parasitized with 86.7% (144/166) of infected birds showing exo-erythrocytic tissue stages in this organ, followed by the lung with 65.0% (93/143) and the brain with 61.0% (100/164)

The distribution of exo-erythrocytic stages in different tissues ranged among individuals from single affected organs to a generalized occurrence. Regardless of the parasite lineage, certain organs exhibited exo-erythrocytic stages more frequently than others (Fig. 1). The spleen was most commonly affected with a rate of 86.7% (144/166), followed by lung (65.0%, 93/143) and brain (61.0%, 100/164) (Fig. 1). In about 50% of the cases, tissue meronts were detected in the heart (87/168), liver (80/152), gizzard (68/129) and bursa of Fabricius (13/25), whereas kidney (40.4%, 55/136), intestine (34.7%, 33/95) and skeletal muscle (18.2%, 2/11) were less commonly affected.

**Fig. 2:**
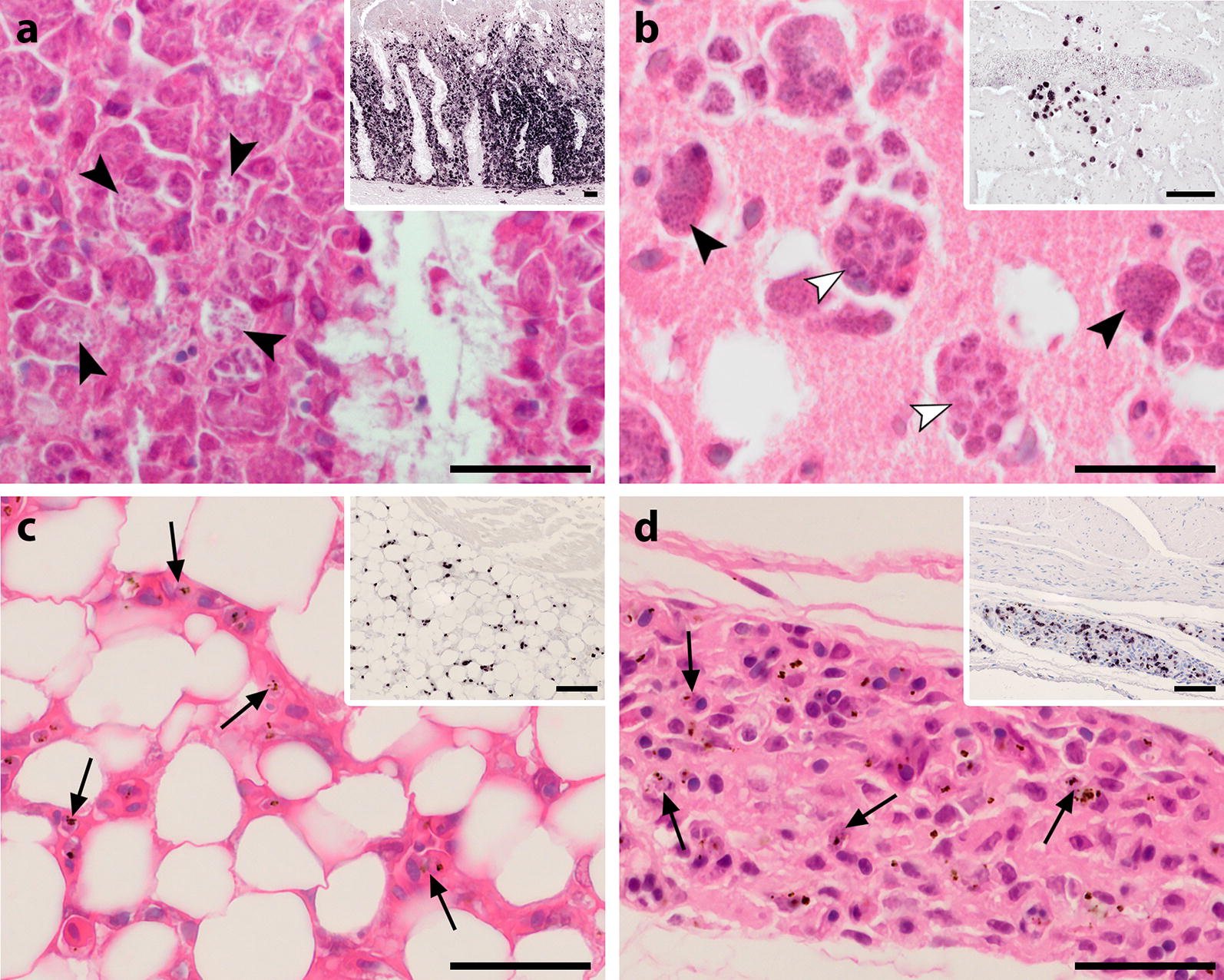
Histological sections of the intestine (**a**), brain (**b**), heart (**c**) and gizzard (**d**) from infected Eurasian blackbirds (*Turdus merula*) showing developmental stages of *Plasmodium matutinum* (**a**, **b**, **d**) and *P. vaughani* (**d**). Parasites were visualized by CISH (**a**–**d**, inserts, low magnification) and identified in haematoxylin–eosin stained tissue sections (**a**–**d**, high magnification). **a** In the intestine, CISH revealed areas of excessive merogony of *P. matutinum* in the intestinal mucosa (insert). Numerous meronts of *P. matutinum* (arrowheads) were located in the cytoplasm of large cells (presumably macrophages) of the lamina propria mucosae accompanied by severe displacement and atrophy of crypt structures. **b** In the brain, clusters of meronts were occasionally observed in several birds, here showing meronts of *P. matutinum* located close to a large vessel (insert). Some of the meronts showed multiple merozoites-containing compartments (white arrowheads). **c**, **d** In a number of birds, abundant small CISH signals were detected in fat tissue (**c**, insert) and the subserosal layers of visceral organs (**d**, insert). In the corresponding HE-stained sections, erythrocytic parasite stages (arrows) were observed to accumulate in these tissues. *Scale bars*: 25 µm, inserts: 50 µm

Evaluation of histological lesions associated with the presence of meronts was difficult because tissues of the submitted dead birds showed varying degrees of autolysis and freezing artefacts. Histological alterations such as necrosis and inflammatory cell infiltration were most often observed in spleen, liver, lung, bursa of Fabricius and intestine and were in some cases associated with excessive exo-erythrocytic merogony of the parasites (Fig. 2a). Apart from ‘regular’ tissue stages, some peculiar observations were made during histological examination of tissues. In a few *P. matutinum* and *P. vaughani*-infected birds, clusters of several exo-erythrocytic meronts were noticed in the brain. Some of the meronts had an untypical appearance as they seemed to divide into multiple smaller parts of similar size, which contained numerous merozoites (Fig. 2b). Another frequent observation was the accumulation of erythrocytic parasite stages of *P. matutinum* and *P. vaughani* in the microvasculature of fat tissue and the serosal layers of visceral organs, most frequently in the heart and the gastrointestinal tract (Fig. 2c, d).

**Fig. 3:**
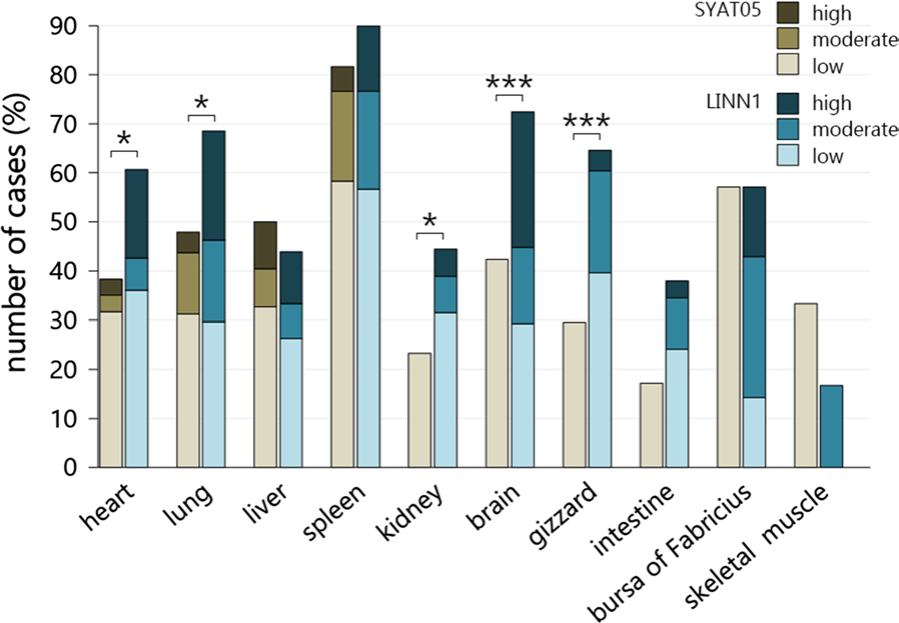
Comparison of exo-erythrocytic parasite burden in different organs of Eurasian blackbirds (*Turdus merula*) infected with *Plasmodium matutinum* LINN1 (n = 65) and *P. vaughani* SYAT05 (n = 62). Birds infected with *P. matutinum* LINN1 showed more frequently moderate- and high-grade parasite burden in almost all organs compared to birds infected with *P. vaughani* SYAT05 (Fisher’s exact: heart, lung, liver, spleen, kidney, brain, gizzard, intestine), *p < 0.05, ***p < 0.001)

To evaluate whether the extent of exo-erythrocytic parasite burden depended on parasite lineage, birds with single infections of the two most prevalent lineages *P. matutinum* (*n* = 65) and *P. vaughani* (*n* = 62) were compared. Because of the low number of song thrushes in the data set and to rule out host species as a confounding factor, only blackbirds were included in this analysis. The *P. matutinum*-infected group showed higher percentages of moderate and high-grade parasite burdens in all organs compared to the *P. vaughani*-infected group with a significant difference found for the heart, lung, kidney, brain and gizzard, but not the liver, spleen and intestine (Fisher’s exact, heart (*n* = 121): *p* = 0.016; lung (*n* = 102): *p* = 0.026; kidney (*n* = 97): *p* = 0.043; brain (*n* = 117): *p* < 0.001; gizzard (*n* = 92): *p* < 0.001; liver (*n* = 109): *p* = 0.896; spleen (*n* = 120): *p* = 0.30; intestine (*n* = 64): *p* = 0.066) (Fig. 3). In *P. vaughani*-infected birds high-grade parasite burden was noticed only in the heart, lung, liver and spleen, whereas in *P. matutinum*-infected birds high-grade parasite burden was observed in almost all organs, with brain (25%, 16/64), lung (22%, 12/59) and heart (18%, 11/67) being most frequently affected.

Morphologically, tissue stages of *P. matutinum* and *P. vaughani* were indistinguishable. In the spleen, liver and bursa of Fabricius, meronts were mostly roundish to oval, while tissue stages in the brain and lungs were often elongate, taking the shape of capillaries. Meronts in the cardiac and skeletal muscle were often slender and oval-shaped. Microphotographs of exo-erythrocytic meronts in different organs are illustrated in Additional file 4 (*P. matutinum*) and Additional file 5 (*P. vaughani*).

According to the PCR and sequencing results, 19 birds (all Eurasian blackbirds) harboured co-infections with both *P. matutinum* LINN1 and *P. vaughani* SYAT05. Using the general *Plasmodium* probe, CISH detected exo-erythrocytic stages in 17 of these birds, showing moderate to high parasite burdens in twelve cases and low parasite loads in five birds. Application of probes specific for either *P. matutinum* or *P. vaughani* to discern their relative roles in co-infections yielded ambiguous CISH-results. Meronts of *P. matutinum* were detected in all 17 cases, whereas CISH using the *P. vaughani*-specific probe resulted in only eight positive cases, seven of which exhibited tissue stages. In three of the seven cases that yielded positive signals with both probes, meronts of *P. vaughani* and *P. matutinum* were equally abundant with few differences regarding tissue distribution, whereas in four cases meronts of *P. vaughani* were more abundant in most organs. Interestingly, in almost half of the cases, the overall abundance of meronts detected by both species-specific probes was lower compared to the general *Plasmodium* probe, indicating lower sensitivity of the species-specific probes.

Birds with single infections of other *Plasmodium* lineages showed different degrees of exo-erythrocytic parasite burdens in their organs. Three blackbirds infected with *P. matutinum* AFTRU05 exhibited high, moderate and low parasite burdens, respectively. Two blackbirds infected with *Plasmodium* sp. TUMER12, a lineage similar to LINN1, showed high and moderate parasite burdens in lungs and spleen, respectively. One song thrush infected with *Plasmodium* sp. TUPHI09, another lineage similar to LINN1, showed single exo-erythrocytic meronts in the lungs and spleen. One blackbird infected with *Plasmodium* sp. TUMER16, a lineage similar to SYAT05, had single meronts in the spleen. The blackbird infected with *P. elongatum* GRW06 showed few meronts in the spleen, liver, gizzard and intestine. The song thrush infected with *P. circumflexum* TURDUS1 was negative by CISH.

### Detection of *Haemoproteus* parasites

In nine of 15 *Haemoproteus*-infected birds, parasites were detected by CISH. In all cases, signals were few in number and restricted to larger vessels and capillaries of different organs, corresponding to blood stages of the parasites, indicating low parasitaemia. In none of the cases, exo-erythrocytic meronts were observed.

### Detection of *Leucocytozoon* parasites

**Fig. 4:**
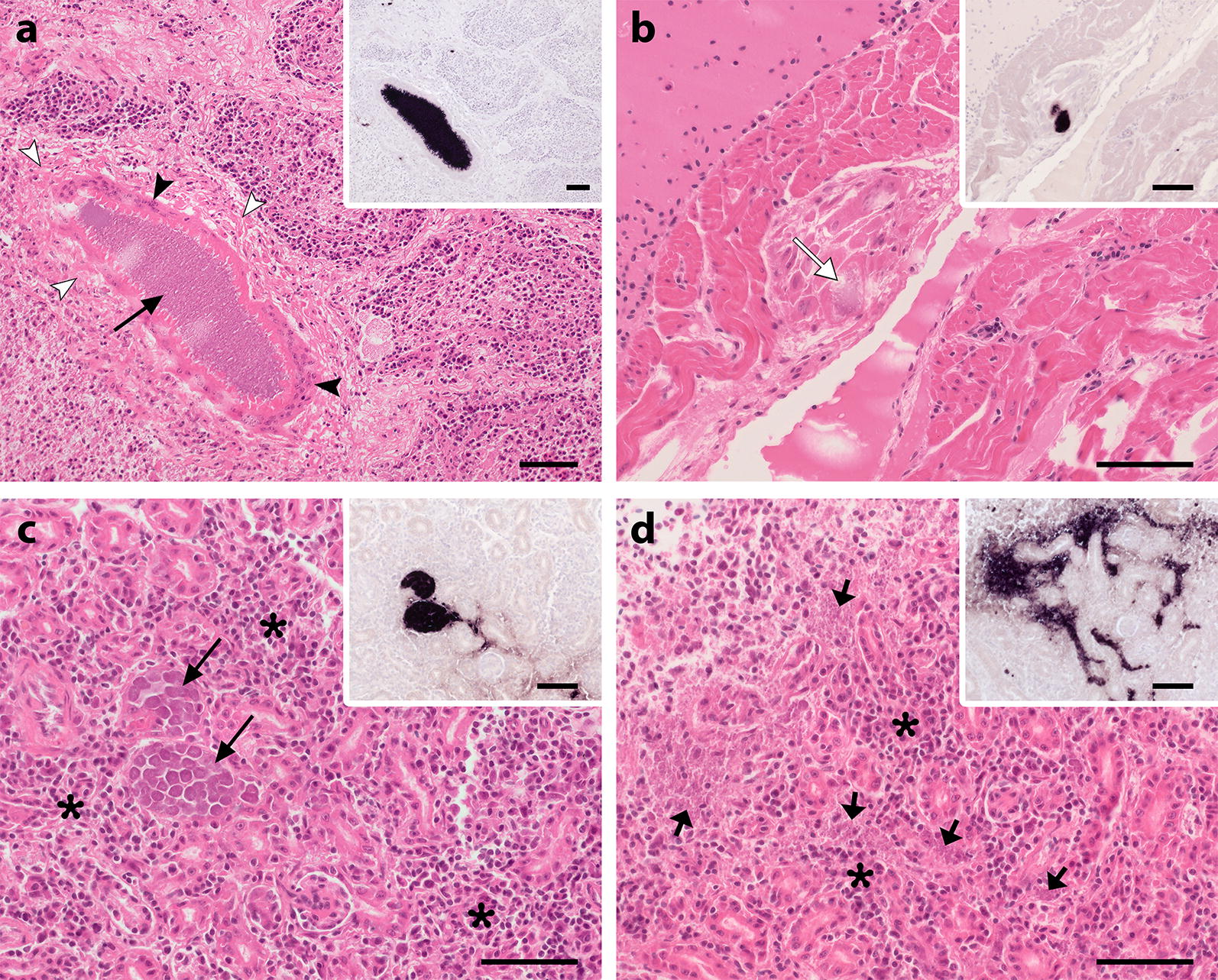
Tissue stages of *Leuocytozoon* sp. TUMER01 in histological sections of two Eurasian blackbirds (*Turdus merula*) detected by CISH (inserts **a**–**d**) and identified in HE-stained sections of the bursa of Fabricius (**a**), heart (**b**) and kidney (**c**, **d**). Megalomeronts observed in the bursa of Fabricius (**a**) and kidney (**c**) consisted of densely packed homogenous masses of merozoites (**a**), surrounded by cells of unclear origin (arrowheads) or featured cytomeres enclosed by a thin wall (**c**). In the heart (**b**), a mature meront featuring merozoites and a defined wall was identied (white arrow). In the kidney, numerous merozoites of 1–2 µm (short arrows), barely identifiable in HE-stained sections, were located in the interstitial tissue, resulting in a strong, scattered CISH signal between renal tubules (**d**). Histopathological changes such as fibrosis (**a**, white arrowheads) and non-suppurative inflammation (**c**, **d**, asterisks) were associated with the parasite stages. *Scale*-*bars*: 50 µm

CISH revealed parasite stages in all *Leucocytozoon*-infections with exception of birds infected with *Leucocytozoon* sp. ASOT06 (three birds). In most cases, however, only blood stages were detected. Two of seven blackbirds that were infected with *Leucocytozoon* sp. TUMER01 showed tissue stages in various organs (Fig. 4). In one bird, megalomeronts were detected in the bursa of Fabricius and kidney. Some of them appeared as densely packed homogenous masses of merozoites which were enclosed by thin capsules and surrounded by cells of unclear origin (Fig. 4a). In the other bird, several megalomeronts featuring cytomeres were detected in the kidney (Fig. 4c). In addition to intact megalomeronts, focal accumulations of numerous merozoites were observed in the interstitial tissue (Fig. 4d). In the HE-stained sections, inflammatory reactions and necroses were associated with tissue stages of the parasites.

## Discussion

This study investigated natural haemosporidian infections in deceased thrushes from Austria, building on the observations of an earlier study which demonstrated an association of passerine bird mortalities with infections of *Plasmodium* spp. [25]. By using a combined molecular genetic and histological approach, the aim was to detect parasites of the genera *Plasmodium*, *Haemoproteus,* and *Leucocytozoon* in Eurasian blackbirds and song thrushes and to determine the burden of exo-erythrocytic stages in their tissues and associated histological effects.

The study showed that Eurasian blackbirds and song thrushes were frequently infected with haemosporidian parasites, particularly with *Plasmodium* species. Among blackbirds, the lineages *P. matutinum* LINN1 (belonging to the subgenus *Haemamoeba*), and *P. vaughani* SYAT05 (subgenus *Novyella*) were predominant, while in song thrushes *P. matutinum* LINN1 was most prevalent. High *Plasmodium* infection rates have been reported in these host species not only from Austria [25], but also from other localities in Europe [24, 35–38] and in thrushes introduced to New Zealand [39], suggesting that these birds are natural reservoir hosts for *Plasmodium* parasites. In contrast to the study by Dinhopl et al. [25], which detected only single infections of parasites belonging to *Plasmodium* spp., the present investigation further showed low prevalences of *Haemoproteus* spp. and *Leucocytozoon* spp. and a mixed infection rate of 20.6%. The use of different PCR protocols and a larger sample size in this study probably explain the contrasting results. While the primers used in the study by Dinhopl et al. target only *Plasmodium* and *Haemoproteus* parasites, the primers and a nested PCR protocol used here allow detection of all three haemosporidian genera. It should be noted, that the PCR protocol by Hellgren et al. [30] has been shown to underestimate the rate of mixed infections in naturally infected birds [40, 41] which might also be the case in this study. Because microscopy of blood smears was not possible and the use of other PCR protocols were beyond the scope of this work, the prevalences reported here should be regarded with caution.

This investigation demonstrates that Eurasian blackbirds were regularly (up to 30%) affected by severe burdens of exo-erythrocytic stages of *Plasmodium* spp. in their organs. As several cases showed, high burdens of tissue meronts were associated with histologic lesions, indicating that severe multiplication of the parasites causes deleterious effects on tissues. These findings are in accordance with the observations by Dinhopl et al. [25] in several native passerine bird species, emphasizing the pathogenic potential of *Plasmodium* parasites in natural hosts. Besides confirming earlier findings, the present study further revealed that the degree of parasite burden was associated with parasite lineage, as shown by the comparison of Eurasian blackbirds infected with either one of the two most prevalent lineages, *P. matutinum* LINN1 and *P. vaughani* SYAT05. In contrast to *P. vaughani*-infected birds, in which extensive tissue merogony was rarely detected, *P. matutinum*-infected birds were frequently affected by severe burdens of exo-erythrocytic stages, suggesting differences in virulence of these two lineages. Different strains of *P. matutinum*, which belongs to the subgenus *Haemamoeba*, have been reported to show variable levels of virulence in avian hosts [42], and previous studies already indicated that infection with LINN1 is associated with disease and mortalities [24–26]. In the present study numerous phanerozoites of *P. matutinum* LINN1 were particularly often detected in heart, lung, and brain of birds with high parasite burdens, similar to a previous report of fatal *Plasmodium* infection caused by this lineage [26]. It seems likely that the severely affected birds in this study also died due to infection with LINN1, which points out the high pathogenic potential of this lineage in Eurasian blackbirds.

Interestingly, song thrushes infected with *P. matutinum* LINN1 mostly showed low parasite burdens. Only one of eight birds exhibited high amounts of exo-erythrocytic parasite stages in some of the organs. This song thrush harboured a mixed infection with *P. matutinum* LINN1 and *Plasmodium* sp. TUPHI08, a lineage almost identical to LINN1. It appears, that single infections with *P. matutinum* LINN1 in song thrushes are not as virulent as in Eurasian blackbirds, but parasite burden might increase in double-infections. However, this remains speculative, as song thrushes were underrepresented preventing direct comparison of parasite burdens between the two host species.

There is limited knowledge on the pathogenicity of *P. vaughani* SYAT05, but generally, the virulence of members of the subgenus *Novyella*, to which *P. vaughani* belongs, is considered to be low [43]. The findings of the present study provide evidence that infection with *P. vaughani* SYAT05 can result in high parasite burdens in Eurasian blackbirds, however, the low numbers of severely affected birds suggest that this occurs infrequently.

It is important to note, that the infected birds in this study all died naturally, and it remains open, to what extent they suffered from concomitant diseases or other pathogens that might have favoured the severity of malaria infections. Birds of the family Turdidae, in particular Eurasian blackbirds, are highly susceptible to Usutu virus (USUV) infection, and epizootic USUV-associated bird mortalities have been reported from several European countries, including Austria [31, 44–52]. More recent studies reported high USUV and *Plasmodium* co-infection rates in Eurasian blackbirds, suggesting that co-infection increases the likeliness of mortalities in infected birds [24, 44]. In contrast to these reports, in the current investigation, USUV and *Plasmodium* infections were weakly associated. A large number of birds used in this study was collected during an earlier USUV surveillance program and tested for USUV, however, of 161 *Plasmodium*-positive thrushes, only 35 blackbirds showed concurrent USUV infection. More importantly, most USUV-positive birds exhibited very low levels of parasite stages both in the blood and in tissues and birds showing high parasite burdens were all USUV-negative, ruling out USUV co-infection as a major driver of *Plasmodium* parasite burden.

The results showed that co-infections with parasites of the two lineages *P. matutinum* LINN1 and *P. vaughani* SYAT05 were frequent among mixed infections. The presence of multiple parasite lineages in the same host can result in within-host competition leading to increased virulence [53, 54], but the modes of parasite interactions in avian haemosporidian co-infections remain largely unknown. Experimental studies demonstrated that concurrent infection with *Plasmodium* spp. can alter infection dynamics of involved lineages and result in synergistic effects on parasitaemia levels in certain hosts [55]. In the present study, species-specific probes were applied to assess the relative burden of *P. matutinum* and *P. vaughani* in the co-infected Eurasian blackbirds, which yielded ambiguous results. While tissue meronts of *P. matutinum* were seen in all cases, the corresponding parasite stages of *P. vaughani* were detected in only half of the cases, suggesting low *P. vaughani* abundance in these birds. In the double-positive cases, tissue stages of both parasite species were equally abundant, or tissue stages of *P. vaughani* were predominant. Unfortunately, from these results, it is impossible to draw any meaningful conclusions on parasite interactions in these co-infections because of two reasons. First, the apparent lower sensitivity of the species-specific probes compared to the genus-specific probes probably obscured true parasite burdens. The reason for the inconsistent results and the weak robustness of the species-specific probes is difficult to explain, but might relate to poor accessibility of selected rRNA target regions due to the complex higher-order structure of these molecules [56]. The efficiency of hybridization of the probes might be improved by simply targeting a different rRNA region. However, this has to be tested in future studies. Second, and more importantly, any conclusions from the observed patterns are limited by the lack of historical information, e.g., time or order of infection, which might have been completely different in the examined individuals. Ideally, to study the effects of parasite-interaction on the development of involved parasite species during co-infections experimental approaches should be considered. In this context, sensitive species-specific probes could support the study of exo-erythrocytic development of parasites during co-infections in future studies.

As noted in the results section, some remarkable observations were made concerning the appearance and location of parasite stages in several birds infected with *P. matutinum* and *P. vaughani*. Although it was not the scope of this study to further pursue detailed investigation of these findings, they might be relevant in regard to the developmental biology of *Plasmodium* in avian hosts and are worth some comments. First, some exo-erythrocytic meronts seemed to comprise multiple smaller merozoites containing compartments. These substructures strongly resemble cytomeres, portions of developing merozoites that are formed in meronts of *Haemoproteus* and *Leucocytozoon* parasites. However, cytomeres have not been reported in *Plasmodium* parasites so far [27] and detailed examinations are required to rule out that the compartments are the result of co-infection of single host cells with multiple merozoites, each producing a separate meront. Second, erythrocytic parasite stages were found to accumulate in the microvasculature of fat tissue and serosal layers of visceral organs. Sequestration, i.e., adhesion of infected red blood cells to the endothelium of capillaries in certain tissues, including brain, lung, intestine and fat tissue, is a common phenomenon of human and rodent malaria parasites and has been shown to contribute to disease severity [57, 58]. It is plausible, that avian *Plasmodium* parasites share the ability to sequester in the microvasculature, however, this is unexplored in birds. It is worth mentioning that the accumulation of erythrocytic parasite stages became apparent due to the strong signals in CISH-treated sections and would have been missed in HE-stainings. Likewise, because it is difficult to detect infected blood cells in HE-stained tissue sections, the phenomenon of avian *Plasmodium* to retain in certain tissues might have been overlooked in the past.

In contrast to *Plasmodium* spp., parasite burden by *Haemoproteus* and *Leucocytozoon* spp. was low in most birds. Blood stages of *Haemoproteus* and *Leucocytozoon* detected in blood cells of infected thrushes indicate that the parasites completed their development within the host and birds died during chronic infection. Only in birds infected with *Leucocytozoon* sp. ASOT06, parasite stages were not observed. This *Leucocytozoon* lineage has been identified in species of Strigiformes from Europe [34, 59] and is probably host-specific, which explains why the parasites could not establish in the thrushes and suggests that these were accidental infections.

Two blackbirds infected with *Leucocytozoon* sp. TUMER01 exhibited disseminated megalomeront formation in several organs. This lineage has been previously reported in Eurasian blackbirds and clusters with other *Leucocytozoon* lineages found in European hosts [38, 60], but has not been linked to a morphologically described species yet. The megalomeronts looked quite different and appeared to be of different developmental maturity. Importantly, an enlarged host cell nucleus (central body) characteristic for *Leucocytozoon* megalomeronts and which distinguishes them from megalomeronts of the genus *Haemoproteus* [27], was not visible in all stages. It is likely, that the host cell nucleus, which can have central or peripheral location, was missed in the examined histological sections here. This demonstrates that morphological identification of parasites in HE-stained tissue sections is not always feasible by examination of single tissue sections and stresses the importance of checking serial sections or the use of molecular detection methods such as in situ hybridization. The histology of the two blackbirds showed that the megalomeronts were associated with tissue damage and inflammatory reactions in different organs. Mortality of these birds could not be definitely linked to *Leucocytozoon*, because other factors than concurrent USUV-infections that might have attributed to death, were not ruled out. However, the histological findings suggest that these birds suffered from leucocytozoonosis. Notably, blood stages of TUMER01 were found in all infected birds, indicating that the parasites underwent merogony in all of these cases. However, except of the two birds showing megalomeronts, exo-erythrocytic stages were not observed. It might be the case that meronts were scarce in these birds and thus missed by examination of tissue sections, but the results rather suggest that megalomeronts were not formed in these birds. Available data from previous studies indicate that the exo-erythrocytic development of *Leucocytozoon* parasites is flexible and the occurrence of certain stages of the same parasite species can be variable in different host species [27]. The findings from this study demonstrate that *Leucocytozoon* sp. TUMER01 does form megalomeronts in Eurasian blackbirds, but the driving forces of megalomeront formation and whether these stages always appear during regular development remain unclear. To understand the role of megalomeronts in the life cycle of *Leucocytozoon* parasites and their pathogenicity in common hosts, further research is needed.

## Conclusion

The present study provides insight into haemosporidian exo-erythrocytic parasite burden in naturally infected Eurasian blackbirds and song thrushes native to Austria. The findings suggest that *P. matutinum* LINN1, a common lineage among thrushes, regularly causes high exo-erythrocytic parasite burdens in Eurasian blackbirds, which may result in disease and mortalities pointing out its pathogenic potential. The low parasite burdens in song thrushes infected with LINN1 illustrate that the same parasite lineage can show different levels of virulence in related bird species, which should be considered when assessing the pathogenicity of haemosporidian parasite species. Finally, the study provides evidence of virulent *Leucocytozoon* sp. TUMER01 infections in two Eurasian blackbirds due to disseminated megalomeront formation. Because little is known about the role of megalomeronts in *Leucocytozoon* in general, more research on the exo-erythrocytic development is needed to understand the pathogenicity of these stages.

## Supplementary information

**Additional file 1.***Plasmodium* parasite stages visualized by the dark-purple CISH-signal in a histological section of the liver. Erythrocytic and exo-erythrocytic stages were distinguished by the size, shape and location of the signals: blood stages showed roundish to oval signals usually not larger than blood cells and were located in capillaries and larger vessels (arrowheads), whereas signals of tissue stages usually exceeded the size of blood cells and showed variable shapes (arrows). *Scale bar*: 20 µm.

**Additional file 2.** Representative photographs showing low- (left), moderate- (middle) and high-grade (right) exo-erythrocytic parasite burden in histological sections of the brain (a–c) and the spleen (d–f) determined by chromogenic in situ hybridization. *Scale bar*: 100 µm.

**Additional file 3.** Comparison of exo-erythrocytic parasite burdens of *Plasmodium*-infected Eurasian blackbirds (*Turdus merula*) and song thrushes (*Turdus philomelos*) collected during different months from May to March of the years 2002–2018.

**Additional file 4.** Exo-erythrocytic meronts of *P. matutinum* LINN1 in haematoxylin–eosin-stained tissue sections of the heart (a–c), lung (d–f), liver (g–i), spleen (j–l) and brain (m–o) of infected Eurasian blackbirds (*Turdus merula*). *Scale bar*: 10 µm.

**Additional file 5.** Exo-erythrocytic meronts of *P. vaughani* SYAT05 in haematoxylin–eosin-stained tissue sections of the heart (a–c), lung (d–f), liver (g–i), spleen (j–l) and brain (m–o) of infected Eurasian blackbirds (*Turdus merula*). *Scale bar*: 10 µm.

## Data Availability

The dataset supporting the conclusions of this article is included within the article and its Additional files.

## Abbreviations

*cytb*: Cytochrome b
CISH: Chromogenic in situ hybridization
FFPE: Formalin-fixed paraffin-embedded
HE: Haematoxylin-eosin
Tris–HCl: Tris–hydrochloride
SSC: Saline-sodium citrate
NBT: Nitro-blue tetrazolium chloride
BCIP: 5-Bromo-4-chloro-3′-indolyphosphate *p*-toluidine salt
HPF: High-power field
