# Supplementary figures and images for: Haemosporidioses in wild Eurasian blackbirds (Turdus merula) and song thrushes (T. philomelos): an in situ hybridization study with emphasis on exo-erythrocytic parasite burden
Source: Malar J. 2020 Feb 12;19:69. doi: 10.1186/s12936-020-3147-6 (PMC7017459; doi:10.1186/s12936-020-3147-6)

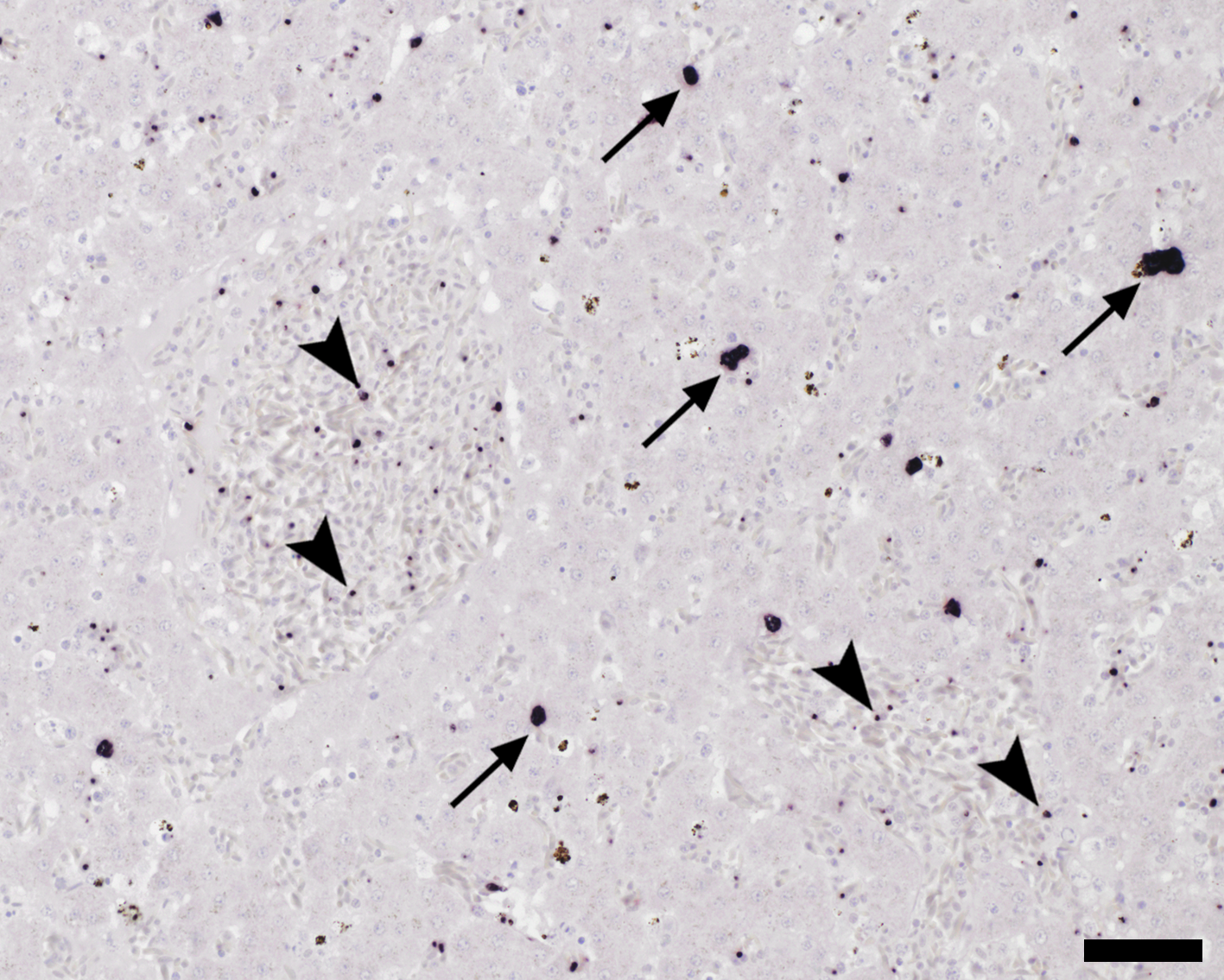

Supplement: Supplementary file 1 — Additional file 1.Plasmodium parasite stages visualized by the dark-purple CISH-signal in a histological section of the liver. Erythrocytic and exo-erythrocytic stages were distinguished by the size, shape and location of the signals: blood stages showed roundish to oval signals usually not larger than blood cells and were located in capillaries and larger vessels (arrowheads), whereas signals of tissue stages usually exceeded the size of blood cells and showed variable shapes (arrows). Scale bar: 20 µm. [file 12936_2020_3147_MOESM1_ESM.tif]

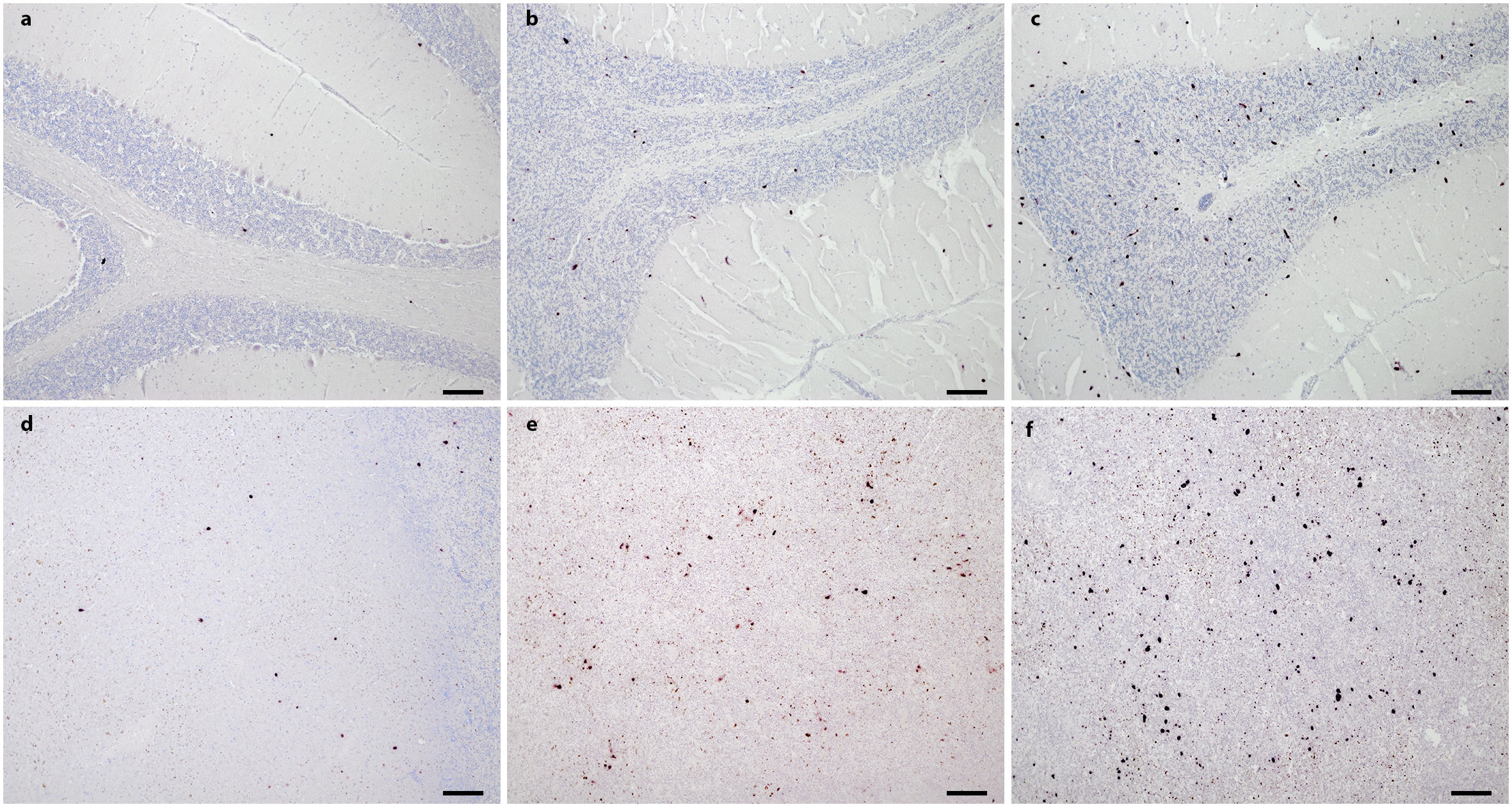

Supplement: Supplementary file 2 — Additional file 2. Representative photographs showing low- (left), moderate- (middle) and high-grade (right) exo-erythrocytic parasite burden in histological sections of the brain (a–c) and the spleen (d–f) determined by chromogenic in situ hybridization. Scale bar: 100 µm. [file 12936_2020_3147_MOESM2_ESM.tif]

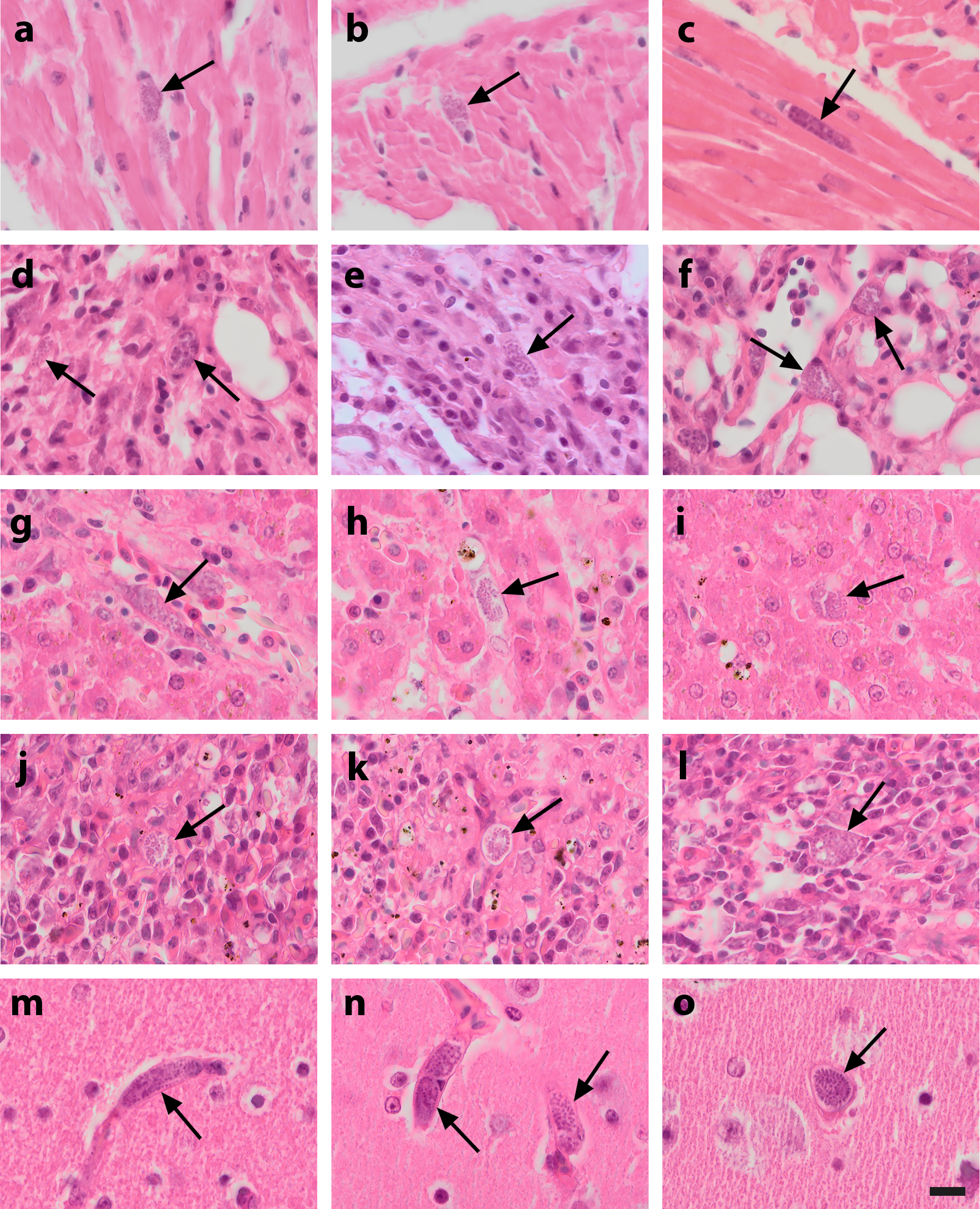

Supplement: Supplementary file 4 — Additional file 4. Exo-erythrocytic meronts of P. matutinum LINN1 in haematoxylin–eosin-stained tissue sections of the heart (a–c), lung (d–f), liver (g–i), spleen (j–l) and brain (m–o) of infected Eurasian blackbirds (Turdus merula). Scale bar: 10 µm. [file 12936_2020_3147_MOESM4_ESM.tif]

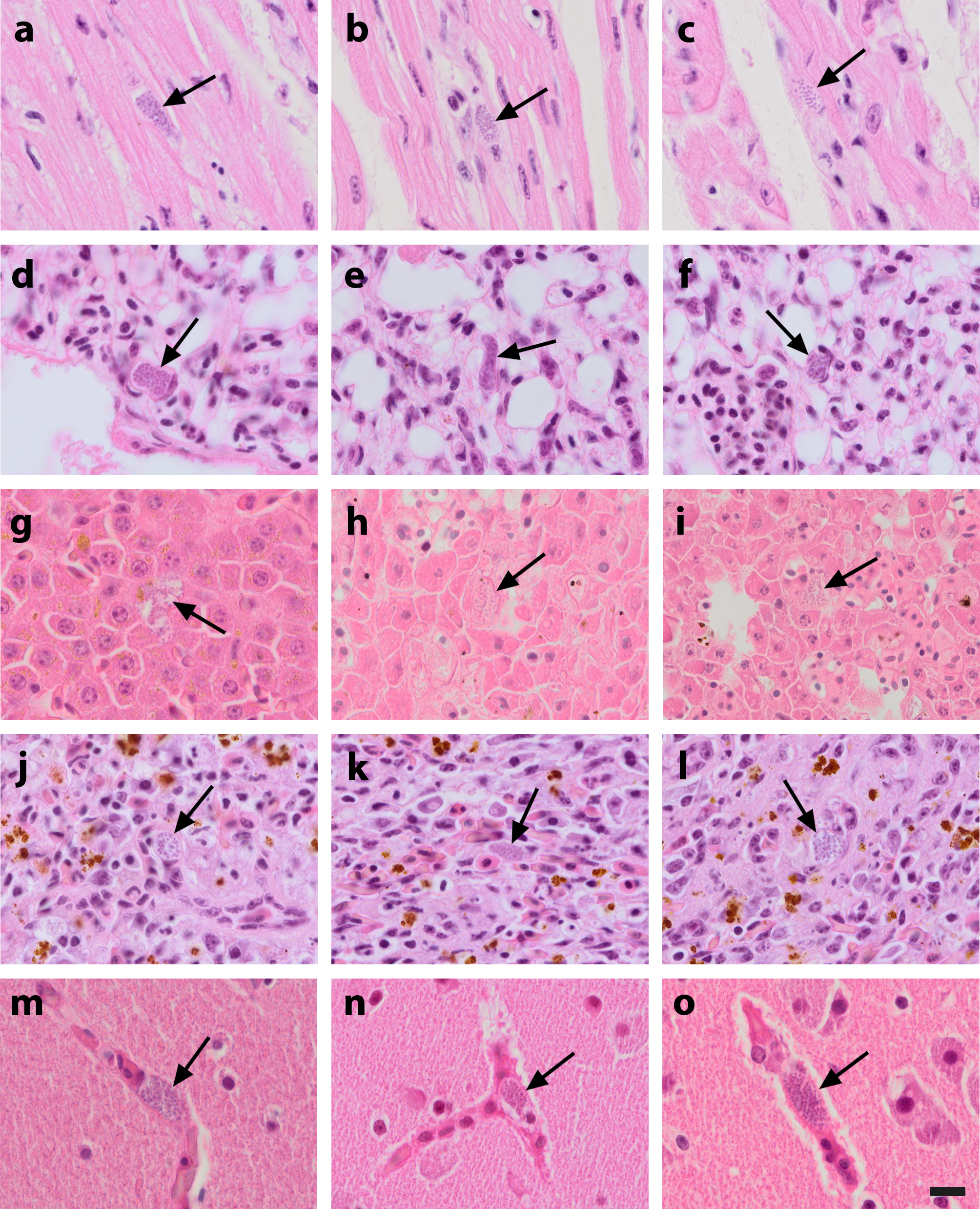

Supplement: Supplementary file 5 — Additional file 5. Exo-erythrocytic meronts of P. vaughani SYAT05 in haematoxylin–eosin-stained tissue sections of the heart (a–c), lung (d–f), liver (g–i), spleen (j–l) and brain (m–o) of infected Eurasian blackbirds (Turdus merula). Scale bar: 10 µm. [file 12936_2020_3147_MOESM5_ESM.tif]
